# Use of aripiprazole long-acting injectable release as a stabiliser. About a case

**DOI:** 10.1192/j.eurpsy.2024.903

**Published:** 2024-08-27

**Authors:** A. Izquierdo De La Puente, P. del Sol Calderón, R. Fernandez Fernandez, M. V. da Silva, M. Garcia Moreno

**Affiliations:** ^1^Psychiatry, Hospital Universitario Puerta de Hierro de Majadahonda, Majadahonda; ^2^Psychiatry, Hospital Universitario Infanta Cristina, Parla; ^3^Psychiatry, CSM San Carlos, El Escorial, Spain

## Abstract

**Introduction:**

A 56-year-old patient diagnosed with bipolar affective disorder type II, who remains stable, with no manifest episodes, thanks to aripiprazole 60mg daily.

**Objectives:**

The aim is to carry out a brief review of the use of the drug as the only stabiliser in bipolar affective disorder.

**Methods:**

A 56-year-old patient, who has been suffering from episodes of hypomania since the age of 40, with episodes of depression. After poor tolerance to the use of the usual stabilisers, and the impossibility of using antidepressants due to hypomanic swings, it was decided to start treatment with aripiprazole orally, up to a maximum of 60mg daily. Despite the fact that the patient, with this treatment, had no side effects and remained more stable psychopathologically, the patient did not comply adequately with the correct dosage, due to his rotating work shifts. This fact explained that although he acknowledged an improvement, he continued with episodes of depressive symptoms lasting several days followed by episodes of hypomanic characteristics.

**Results:**

For this reason, it was decided to change treatment to aripiprazole long-acting injectable, in order to ensure linear blood levels of the drug. Initially, it was decided to prescribe 400mg every 28 days. However, after the first administration, 20 days later, the patient began to show dysphoric mood, with marked emotional lability, living in an egodystonic manner. For this reason, the dose was increased to 600mg on a monthly basis. Since then, after a year and a half with the same treatment, the patient has been stable and in line. There has been no further decompensation of the underlying psychopathology and no side effects.

**Conclusions:**

Aripiprazole in TAB is superior to placebo in type I patients, mainly affecting manic and mixed episodes, but not so much in depressive episodes. It has also been observed that it not only acts in the acute phases, but also has a stabilising function, preventing manic episodes.

One study showed that up to 65% of patients on oral aripiprazole in whom it was replaced by AOM remained clinically stable. In the same study, approximately 50% of those who completed 52 weeks of follow-up were able to maintain clinical stability.

**Disclosure of Interest:**

None Declared

